# Data-poor categorization and passage retrieval for Gene Ontology Annotation in Swiss-Prot

**DOI:** 10.1186/1471-2105-6-S1-S23

**Published:** 2005-05-24

**Authors:** Frédéric Ehrler, Antoine Geissbühler, Antonio Jimeno, Patrick Ruch

**Affiliations:** 1 Artificial Intelligence Laboratory, University of Geneva, Geneva, Switzerland; 2 Medical Informatics Service, University Hospitals of Geneva, Geneva, Switzerland; 3 CERN, Geneva, Switzerland

## Abstract

**Background:**

In the context of the BioCreative competition, where training data were very sparse, we investigated two complementary tasks: 1) given a Swiss-Prot triplet, containing a protein, a GO (Gene Ontology) term and a relevant article, extraction of a short passage that justifies the GO category assignement; 2) given a Swiss-Prot pair, containing a protein and a relevant article, automatic assignement of a set of categories.

**Methods:**

Sentence is the basic retrieval unit. Our classifier computes a distance between each sentence and the GO category provided with the Swiss-Prot entry. The Text Categorizer computes a distance between each GO term and the text of the article. Evaluations are reported both based on annotator judgements as established by the competition and based on mean average precision measures computed using a curated sample of Swiss-Prot.

**Results:**

Our system achieved the best recall and precision combination both for passage retrieval and text categorization as evaluated by official evaluators. However, text categorization results were far below those in other data-poor text categorization experiments The top proposed term is relevant in less that 20% of cases, while categorization with other biomedical controlled vocabulary, such as the Medical Subject Headings, we achieved more than 90% precision. We also observe that the scoring methods used in our experiments, based on the retrieval status value of our engines, exhibits effective confidence estimation capabilities.

**Conclusion:**

From a comparative perspective, the combination of retrieval and natural language processing methods we designed, achieved very competitive performances. Largely data-independent, our systems were no less effective that data-intensive approaches. These results suggests that the overall strategy could benefit a large class of information extraction tasks, especially when training data are missing. However, from a user perspective, results were disappointing. Further investigations are needed to design applicable end-user text mining tools for biologists.

## Introduction

Numerous techniques help researchers locate relevant documents in an ever-growing mountain of scientific information. Next, it becomes important to develop tools able to help people process this data for use in digital libraries and electronic databases (see [1] for a survey). The BioCreative intitiative, a joint evaluation campaign organized by the Centro Nacional de Biotecnologia (CNB) and the MITRE and supported by the European Molecular Biology Organization (EMBO), aimed at explored the application of text mining tools to support annotation of molecular biology databases. Four different types of tasks were proposed:

• Gene and protein named entity boundary detection (task 1a). This is a classical task in information extraction, and has been largely investigated in the context of MUC [2] conferences as well as more recently in more biomedical fori, such as the JNLPBA workshop shared task proposed this year for at COLING .

• Passage retrieval (tasks 2.1). The task in well-known in question-answering [3]. The point of this task is to retrieve a short passage rather than a complete document.

• Text categorization (tasks 1b, 2.2). In task 1b, the targeted categories are a set of gene and protein names, while in task 2.2, the categories are the terms listed in the Gene Ontology (GO). For task 2.2, the passage supporting the annotation is also to be provided (task 2.1).

• *Ad hoc *information retrieval (tasks 2.3 and 2.4). These two tasks were discarded due to the lack of participants.

Our participation focused on task 2.2, which also includes task 2.1. From a functional point of view, task 2.1 is defined as follows: given a Swiss-Prot triplet, i.e. a protein, a GO term and a related article, participants had to extract a short passage that substantiates selection of a GO a category. Task 2.2 is more complex: given a Swiss-Prot pair, containing a protein and a relevant article, participants had to automatically assign a set of GO categories, then for each of the assigned GO categories, we located the appropriate passage, which supported the attribution of the GO term. The experimental design assumes that the number of GO categories assigned for each protein is known a priori.

The plan of the paper is the following: introduction of the background research supporting our work in section 2; description of the data sets and the architecture of the system in section 3; results, the official evaluation merged with evaluations made after the competition, in section 4; conclusion and future works, in section 5.

## Background

In this section, we relate the content of the paper to the state-of-the-art. Both passage retrieval and automatic text categorization are introduced, however as the rest of the paper, which reflects the second BioCreative task, the presentation focuses on the categorization task.

### Passage retrieval

Passage retrieval is an important step in question-answering (QA). It bridges the gap between document retrieval and very short textual answers needed for QA. However, the purpose of the passage retrieval task proposed in BioCreative is to find a short fragments which would appropriately support 1) the already known GO annotation in task 2.1, and 2) the automatic GO term assignement in task 2.2. In both cases the targeted text is already known. Thus, the task is similar to the *known-item search *task [4]. In TREC, this task aimed at retrieving s single know document in corrupted collections. Corruptions were caused by misspellings [5] or by running optical character recognition [6]) tools.

### Text categorization

Text Categorization (TC) aims at attributing a set of concepts to an input text. Typical applications use a set of keywords to be selected into a *glossary*. TC is performed daily by professional indexers working in digital or classical libraries. However, keyword assignment is only a particular instance of text categorization. TC can also be seen as an information extraction task, when conducted for named-entity (NE) recognition purposes as investigated in task 1b. Computer-based concept mapping technologies include:

• *retrieval based on string matching*, which attributes concepts to texts based on shared features (words, stems, phrases...);

• *empirical learning of text-concept associations *from a training set of texts and their associated concepts.

In the former approach, the targeted concepts are indexed. Each indexing unit is attributed with a specific weight. While in the latter, a more complex model of the data is built in order to provide text-concept associations beyond strict features sharing. Retrieval based on string-matching is often presented as the weaker method [7] of the two, but in many real situations, like those defined in the BioCreative challenge, learning approaches cannot be applied. For instance, empirical learning methods require large training sets of data that are usually not available and whose development costs would exceed the budget of most research groups. Additionally, the size of category sets can be some orders of magnitude above the capacities of current learning algorithms running on a standard computing framework. Designing TC as a retrieval task means indexing of a collection of terms, in our case terms from the GO, as if they were documents, and then processing each document as if it was a query. Then, the retrieval tool uses the score attributed to each term to rank them. Because the document collection is made of entities (terms in a controlled vocabulary) that are clearly shorter than usual documents. Our study aims at exploring the behavior of classical statistical models. For TC, the use of a vector space engine, using both stems and linguistically-motivated indexing features, and its combination with a search tool based on pattern matching constitutes the main modules of our system. We also investigated some refinements of this core combination.

#### Scalability issues

Automatic text categorization has been extensively studied and has led to an impressive number of papers. A partial list (see  for an updated bibliography) of machines learning approaches applied to text categorization includes naive Bayes [8], support vector machines [9], boosting [10], and rule-learning algorithms [11]. However, most of these studies apply text classification to a small set of classe, usually a few hundreds, as in the Reuters' collection [12]. In comparison, our retrieval methods are designed to handle large class sets since they relies on an inverted file to allow fast categorization. The inverted file relates each indexing unit (word or stem) to the terms where it occurs in the GO. The size of the inverted file, which additionally stores the weight of each word (or stem), is an important parameter but 10^5–6 ^is still a modest range so that even large controlled vocabularies can be indexed.

In text categorization based on learning methods, the scalability issue is twofold. It concerns both the ability of these data-driven systems to work with large concept sets and their ability to learn and generalize regularities for rare events. Theoretically, if large multi class problems can be recast as binary classifiers in order to be solved by learning approaches, in practice it is often difficult. Larkey and Croft [13] show how the frequency of concepts in the collection is a major parameter. Our approach is data-poor because it only demands a small collection of annotated texts for fine tuning as opposed to data-intensive machine learning approaches, which require large annotated sets.

To our knowledge the largest set of categories ever used by text classification systems is above 10^4^. These systems were applied to the domain of life sciences. Yang and Chute [14] worked with the International Classification of Diseases (about 12000 concepts). Similarly, the OHSUMED collection contains 14301 Medical Subject Headings (MeSH). In contrast, our system is tailored to be applied to much larger class sets. The Unified Medical Language System (UMLS) contains 871,584 different concepts and 2.1 million terms (with synonyms), while TrEMBL contains about 700,000 protein names, often including synonyms. For the BioCreative competition, the categorization space of our system was restricted to the GO partition of the Unified Medical Language System (UMLS).

#### Indexing units

In addition, to usual word-based features more elaborated indexing units have been proposed in information retrieval (IR). The general idea in indexing entities, which are different than words (or stems), is to handle information as conveyed in word collocations. Thus, expressions such as *cystic fibrosis *can be seen as one semantic entry in an inverted file. Various phrase indexing methods have been proposed in the past and generally, retrieval or categorization performance conclusions on the use of phrases as indexing units were inconsistent [15]. For IR, Hull et al. [16] and Strzalkowski et al. [17] used phrases and were able to report some improvement. For text categorization, Tan et al. [18] and Mongovi et al. [19] have reported that statistical bigrams increased performance, while Toole and Chen [20] relied on linguistically-motivated phrases. Mitra et al. [21] re-examined the use of statistical and syntactic phrases for retrieval and came to the conclusion that "once a good basic ranking scheme is used, the use of phrases do not have a major effect on precision at high ranks". For linguistically-motivated phrases, Arampatzis et al. [22] question the use of syntactic structures as substitute for semantic content. As for our present concerns, statistical phrase indexing is problematic. Usually inspired by mutual information measures [23], it requires important volumes of training data, while we aim at designing a data independent system. Therefore, in our systems phrases are based on syntactic parsing [24] rather than statistical analysis. However, let us remark thaz data needed to identify statistical phrases are not of the same kind as those needed for training a classifier: the former approach requires only large corpora, while the latter needs supervision, i.e. annotated data, so both tasks are data-intensive but discovering statistical phrase extraction is much cheaper than text categorization.

## Methods

Most data sets and metrics are common to each of the subtasks, therefore we introduce these aspects first, then the methods used for conducting each task are reported.

### Resources

The data resources used in the experiments can be separated into three subsets, the document collection, the Swiss-Prot [25] records and the GO terms [26]. The Gene Ontology merges three structured vocabularies, organized as ontologies, that describe gene products in terms of their associated biological process, cellular component (1368) and molecular function in a species-independent manner. The molecular function terms describe activities at the molecular level. A biological process is accomplished by one or more ordered assemblies of molecular functions. The cellular component is a component of the cell, which is part of some larger object. For example either an anatomical structure or a gene product group.

### Collections and metrics

An initial set of 640 articles (data set "initial", or DSI) from the *Journal of Biological Chemistry*, was provided by the organizers, 320 were used for tuning our tools (DST) and the other half was used for non-official evaluations (data set "non-official", or DSNO). The data set used for the official evaluation of task 2.1 comprised 1048 *proteins-Gene Ontology category *relations (DSO, for "official"). The number of relations depends on each participants, because some participants decided not to submit results for every relations. For 2.2, 661 *proteins-Gene Ontology category *relations were evaluated. However, for tuning our system prior to submitting our official run, as well as for conducting post results investigations, we used retrieval-inspired metrics. Retrieval techniques yield a ranked list of terms for each document, therefore the main evaluation measure is usually based on the mean average precision (MAP). In addition, for GO annotation, the number of token assigned per protein ranges from 1 to 15 (see Table 1), but 90.6% of proteins in the DSI sample of Swiss-Prot have less than 5 GO terms, so that precision for top ranks (*Precision*_*atRecall *= 0 _or mean reciprocal precision), is probably more important; therefore, this metric is mostly used in our non-official evaluations. When MAP is used, the top 5 terms returned by the system are used.

For task 2.1 the expert has to decide whether the evidence text corresponds to the given GO concept and protein, or if it is not appropriate. Additionally, in task 2.2, the judge assesses whether the GO concept has been correctly predicted for each text. There are three different marks (*high*, *generally*, *low*) to evaluate the quality of the results. These marks evaluate GO concept and protein separately. For task 2.1, *high*, *generally *and *low *evaluate the relevance of the sentence, which supports the annotation of the protein with GO concepts. For the task 2.2, *high *means that the protein or the GO concept has been correctly predicted. *Generally*, as an evaluation for the GO term, means that it is not totally wrong but too general to be useful for annotation. *Generally*, as an evaluation for the protein means that the specific protein has not been found but instead a homologue from another organism or a reference to the protein family. *Low *means that the answer was wrong.

### Passage retrieval

The purpose of the passage retrieval task is to facilitate and improve annotation by offering a short segment of text that can indicate the correct GO term. Our approach is based on the idea that the relevant passage and the GO terms share some kind of lexical, and hopefully semantic, similarity. Therefore, the basic method consists of searching for the concept directly in the text. For the passage retrieval task, only the GO term is used to rank passages from the input text. Although, using the protein name and synonyms of the GO term could have been useful to expand the matching power of our approach, we decided to focus on an high precision matching rather than relying on additionnal materials. A possible improvement would be to boost GO concepts, which occur more than once in the candidate list. Identifying parts of GO terms in text is an simple strategy, which does not require any training data set and which can be manually tuned. The main difficulty encountered with this approach is defining a distance that measures the similarity between a GO concept and a given sentence. Different types of distances were tested, but the basic idea is to rank the candidate sentences and to select a single top-ranked passage. Two independendt modules were developped: a sentence splitter, which defines the basic retrieval units, and the sentence ranker. Although specialization of the parameters used for each of the three GO axis could have been beneficial, we used the same settings for each of the three GO axis.

#### Sentence splitting

As preliminary observations we noted that applying our tools on full text articles rather than on abstracts did require improving our pre-processing tools, especially to detect sentence boundaries, therefore the official competition experiments were done using abstracts. For experiments conducted afterwards, the impact of using full-text articles was investigated.

For passage retrieval, the length of the appropriate segment to be considered was crucial. Following what was learned from the information extraction task of the TREC Genomic track, we assumed that sentences were likely to be relevant segments [27]. The TREC task aimed at returning a Gene Reference into Function (GeneRIF), i.e. a short passage, which provides information on the function of a protein in the LocusLink repository.

Lacking clean training data, we decided not to investigate the use of machine learning approaches to solve the sentence pre-processing problem (as in [28]), and instead we decided to use simple manually crafted regular expressions. The tool relies on a set of finite-state automata, which are applied sequentially. Although the system is simple, it offers a certain level of maintainability and a good accuracy (97%), which is similar to more advanced sentence boundary detection methods.

#### Sentence weighting

Two different similarity measures have been used to compute a score between sentences and GO terms. The two similarity measures are: 1) a high precision but low discriminative power exact match method and 2) a low precision but good recall fuzzy string-edit distance. These two measures are then linearly combined to obtain a unique score for each sentence in the input document as in the following equation:



with the following parameters and parameter values:

• *s*_0_: perfect score;

• *s*_1_: fuzzy score;

• *w*_0_: weight of *s*_0_;

• *w*_1_: weight of *s*1.

The direct match method computes a Dice-like distance as in the following equation:



Each time a word of the GO concept is found in the candidate passage the GO term and passage intersection set is increased by one. This score is divided by the total number of words, which composes the concept. The normalization factor is important to smooth length variations in the GO controlled vocabulary. It is also interesting to notice that full and exact match is unusual, but when it occurs (e.g. when a five token GO term if found in the document) then very high precision is achieved, thus precision becomes a trivial issue. In a quite unusual manner for categorization and information retrieval purposes, recall is more difficult to achieve. Indeed, unlike in large text collections (MEDLINE, Web...), where the natural redundancy of information help to find a relevant document whatever words are used to query the system, searching for a relevant passage in an abstract is more challenging regarding recall.

The string edit distance module computes a distance between two strings. The score counts the minimal number of modifications (insertions, deletions and substitutions) needed to transform the first string into the second one (see [29], for a short introduction or [30] and [31], for a comprehensive presentation). String-edit distances operations are very sensitive to small cost variations making this step very time-consuming.

As shown in Table 3, different distances were tested. The Levenstein distance is the basic edit distance. All basic operations, i.e. insertion, deletion and substitution, cost 1. The Levenstein distance computes the score between two strings by selecting at each stage the cheapest operation to transform one string into another. The final score expresses the distance between the two strings. In the Smith-Waterman, a particular cost is associated to each operation. In our experiments, costs were chosen by manual tuning. The Jaro metric measures distances between tokens. It is well adapted to assess distances between two terms, which may share similar tokens in a different order. In the resulting combined distance, transposition between two words are under-weighted as compared to operations made on single characters. The Jaccard distance computes the distance between two sets by the ratio of the size of their intersection to the size of their union.

The choice of the best distances was made empirically. Some characters, such as "-" or digits, have a very low replacement costs. As exemplified in Table 3 given the three following sentences and the term "protein serine/threonine kinase activity", the Smith-Waterman distance performed generally well:

S1. *Cdc42-induced activation of the mixed-lineage kinase SPRK in vivo*.

S2. *Src homology 3 domain (SH3)-containing proline-rich protein kinase (SPRK)/mixed-lineage kinase (MLK)-3 is a serine/threonine kinase that upon overexpression in mammalian cells activates the c-Jun NH(2)-terminal kinase pathway*.

S3. *This is, to the best of our knowledge, the first demonstrated example of a Cdc42-mediated change in the in vivo phosphorylation of a protein kinase*.

In this example, we assume that S2 is the best candidate sentence. Two direct matches are observed in S2 and S3, and so these two segments are better candidates than S1, but to rank segments S2 and S3, we relied on the string-edit distance module. In Table 3, we see that both Smith-Waterman and Jaccard measures are discriminant, while neither Jaro, nor Levenshtein are effective. The final score is a linear combination which favors Smith-Waterman and Jaccard over Jaro and Levenshtein distances. This score will be used in our evaluations to estimate the reliability of the passage assignement.

### GO categorization

In this section, we present the architecture of the GO categorization tool. The weighting schema of the tool will be the same for each of the three classifiers we have developed. Each classifier corresponds to the mutually exclusive axes of the GO: cellular components, molecular functions and biological processes. Two main modules constitute the skeleton of our system: the regular expression (*RegEx*) component, and the vector space (*VS*) component. The former component uses both tokens as indexing units, while the latter uses both stems (Porter) and noun phrases. Each of these basic classifiers uses known approaches to document retrieval. The first tool is based on a regular expression pattern matcher. It is expected to perform well when applied on very short documents such as keywords. As shown in Table 2, 90% of GO terms do not contains more than 5 tokens. The second type of classifier is based on a vector space engine. This second tool is expected to provide high recall in contrast with the regular expression-based tool which should privilege precision. To draw a parallel between the categorization and the passage retrieval task, the pattern-matcher and the exact match measure plays the same role, while the vector-space behaves like a fuzzy and long-distance similarity measure.

#### Regular expressions

Our system does not use any specific string normalization module. The system extracts every contiguous sequence of 5 tokens by moving a window through the abstract. These pentagrams are then matched against the collection of GO terms. Basically, the manually crafted finite-state automata allow two insertions or one deletion within a GO term. Ranking of the proposed candidate terms is based on these two basic edit operations: insertion costs 1, while deletion costs 2. The resulting pattern matcher acts as a term proximity scoring system [15], but with a 5 token matching window. Krallinger and Padron [32] use a similar strategy but they generalize the idea and vary the window size too.

#### Vector space classifier

The vector space module is based on a general IR engine with *tf.idf *(term frequency-inverse document frequency) weighting. We used the SMART [33] representation for expressing statistical weighting factors. Given a collection profile (queries and targets), it is possible to calculate an optimal weighting scheme by varying a set of parameters. The main parameters are provided in Table 4. A retrieval experiment can be characterized by a pair of triples -*ddd.qqq*- where the first triple corresponds to term weighting used for the document collection (hence the symbols *d*), and the second triple corresponds to the query term weight (hence the symbols *q*). Each triple refers to a term frequency, an inverse document frequency and a normalization function as provided in Table 4. More elaborated weighting, such as the *deviation from randomness *[34] and the pivoted normalization [35] were tested but did not result in any improvement as compared to the cosine normalization given in Table 4.

The engine uses stems (Porter, with minor modifications) as indexing units and a stop word list (544 items). We observed that cosine normalization was especially effective for our task. This is not surprising, considering the fact that cosine normalization performs well when all documents have the same length [35].

#### GO thesaurus

An updated version of the GO was released some days before the competition, but all experiments were done with a slightly older version than the one used for establishing the benchmark. It contained 13203 synonyms: 378 for components, 1931 for processes, and 10904 for the function axis. Together with the 16687 terms, chosen as best representative of GO concepts, the index contains 29900 entries. Although working with an updated version could have brought some improvement, it is important to notice that the proposed learning-free approach allows to be largely independent on *concept drifting *issues [36,37], which necessary occur when controlled vocabularies evolve to reflect changes in the field. An example of synonyms for each axis is provided in Table 5. When the thesaurus is used, terms variants are indexed like other terms (preferred terms), but for each set of synonyms, only the best ranked term is kept in the candidate list to avoid duplicating GO concepts.

#### Phrase indexing

GO terms contain between 1 and 28 words and almost verb-free noun phrases (NP) if we omit some rare participle forms such as in "cell-cell signaling *involved *in cell fate commitment", which occur in less than 0.01% of GO terms. Noun phrase indexing was expected to be beneficial because of the profile of these terms. In our approach, only the content of GO terms is stored in the indexes, and phrase recognition is only applied on the input document in order to identify possible GO terms. Formally, this manipulation of the abstract can be viewed as a reformulation process. The abstract is translated into a set of noun phrases before to be matched to the list of GO terms. Our working hypothesis is a weak variant of the *Phrase Retrieval Hypothesis *[22]. We assumed that NP recognition can help reducing *noisy mapping *for *subterms*.

Our shallow parser uses both statistical and manually written patterns, applied at the syntactic level (part-of-speech) of each sentence [24], to identify noun phrase boundaries. The parser concentrates on adjective (A) and noun (N) sequences, such as: [A*] [N*], i.e. N, AN, NN, ANN, NNN, AANN, ANNN, NNNN, AANNN, NNNNN... adjectives as well as prepositions such as *of*, *with *are optional. Unlike in other technical glossaries [38], we observed that templates with conjunctions are rare in GO terms. We counted 1423 occurences of conjunction tokens the GO terminology terminology (i.e., almost 1%), therefore we decided to ignore it.

We call *noisy subterm mapping *an erroneous behavior of the mapping process, when it selects some erroneous GO terms that are part of a relevant term. Thus, considering an input text dealing with the term *cystic fibrosis*, both *cystic *and *fibrosis *are irrelevant subterms likely to be proposed as indexing units, so being able to recognize that *cystic fibrosis *constitutes a noun phrases will help discard these two *noisy *candidates. However, discarding all subterms from the candidate list may result in negative effects, so that subterm removal must be based on contextual evidences. If a subterm occur in the input text as an autonomous noun phrase, then it is kept in the candidate list. Therefore two different indexes (or view of the input text) are constructed. The merger of this index with the index of stems is described in the next paragraph.

#### Fusion of classifiers

The hybrid system combines the regular expression classifier with the vector-space classifier. Unlike Larkey and Croft [13] we do not merge our classifiers by linear combination because the *RegEx *module does not return a scoring consistent with the vector space system. The combination of classifiers uses the list returned by the vector space module as a *reference *list (*RL*) and the list returned by the regular expression module is used as *boosting *list (*BL*). This method serves to improve the ranking of terms listed in *RL*. A third factor takes into account the length of terms. Both the number of characters (*L*_1_) and the number of tokens (*L*_2_, with *L*_2 _> 3) are computed, so that long and compound terms, which appear in both lists, are favored over single single and short terms. We assume that the reference list has good recall, and we do not set any threshold on it. For each concept *t *listed in the *RL*, the combined Retrieval Status Value (*cRSV*, equation 1) is:



The value of the *k *parameter is set empirically.

The index of phrases is used to reorder the set of terms returned by the engine. The strategy is the following: when a given term is found in the list of terms (*TL*) returned by the hybrid system (*RegEx *+ *VS*), and this term is not found alone in the phrase list (*PL*) stored for this abstract, then the *RSV *of this concept is downscored. The shorter the subterm, the more its RSV is affected, as expressed in the following equation, which gives the final RSV. *fRSV *; *m *= 16 in equation 2, since GO terms contain no more than 15 words:



In principle, to transform a retrieval engine, which returns a ranked list of concepts, into a categorization system, which make binary decisions on each concept, it is necessary to set a threshold on the retrieval status value. However, the number of concepts to be returned for each protein-GO axis pair is known, so this threshold may be a priori ignored in the current design of the categorizer.

#### GO definition, prior probability and full article

As shown in Table 6, most GO terms (about 90%) are provided with a definition. This definition can be used to expand the matching features between an abstract and the GO terms in the feature space. However, because features in GO terms are more important than features appearing in definitions, an underweighting factor is applied on features of the definition.

Another refinement that we tested concerns the application of a prior probability. In table 7, we give the distribution of GO term in the DSI data set. The prior probability factor is applied after logistic smoothing on the RSV returned for each GO term. GO categories occurring less than three times are not taken into account.

## Results and Discussion

In this section, we present and discuss the official results as well as results gathered after the competition. All official results were provided by the BioCreative judges.

### Passage retrieval

Official evaluations, distributed over each GO axis, are reported in Table 8. From these data, we can observe that biological processes (710) are more abundant than molecular functions (361) and cellular components (185). This table also confirms that the evaluated set (# evaluated) is a balanced sample of the original data (# passage). In general, we also observe that predictions are more relevant regarding the protein (Prot) than the GO category (GO). The total is 55% vs. 15% respectively for the *high *results. In general, the quality is equally distributed among the three axis regarding the protein, but regarding GO annotation, passage retrieval seems more difficult for biological processes (*high *= 12%; *generally *= 11%) than for cellular components (*high *= 18%) or molecular functions (*high *= 15%) categories. For the passage retrieval task, our system achieved a competitive precision regarding both the GO annotation and the protein annotation (respectively 7.06% and 25.57% for results considered as perfect by the judges) if we consider that the only system [39] which achieves a better protein annotation score (28.86%) perform less effectively regarding the GO annotation (5.44%).

### Gene Ontology Annotation

**Figure 1:**
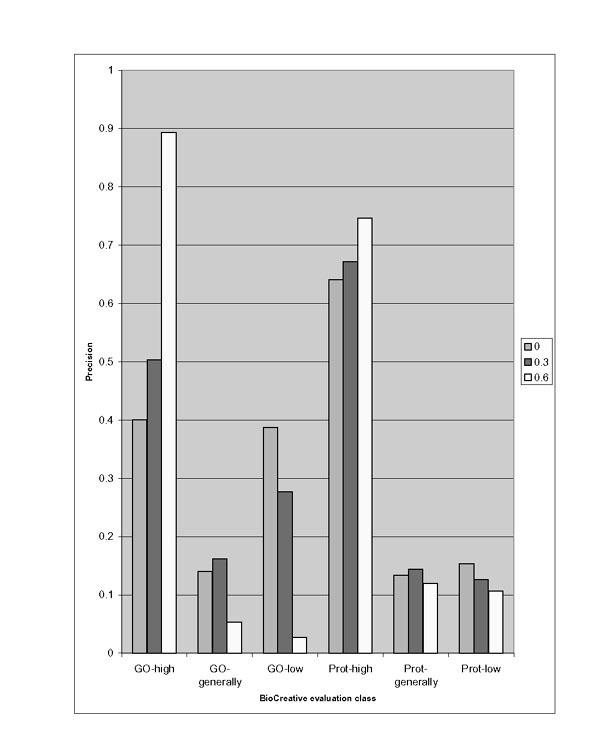
Task 2.2. Official results. Submitted runs are in gray (threshold = 0). White histograms show the performance of the passage retrieval tools when only highly reliable results are considered (threshold = 0.6). Black histograms show the performance of the retrieval tools using an intermediate confidence threshold (0.3).

For task 2.2, official results for all three axis are reported in Figure 1 both for protein (Prot) and GO annotation. In Figure 1, evaluations made by GO curators are provided for different confidence estimations. A confidence threshold of 0 means that all predictions were evaluated. This threshold dictated the official results. Two other thresholds are proposed, 0.3 and 0.6. As expected the passage retrieval score returned by the sentence ranker is an excellent confidence estimator. The trend is true for both *GO *and *Prot*. Strikingly a precision close to 90% is obtained for passages related to the GO annotation, when the normalized similarity is above 0.6. Although precision is impressive, this setting brutally affects recall. Only 75 out of the 889 evaluated passages are selected as correct with such a confidence threshold. We observed that trading recall for precision does not affect the comparative effectiveness of our system. The best high-precision system reports only 80% precision, together with a lower recall. Additionally this result is obtained by submitting only 45 results.

**Figure 2:**
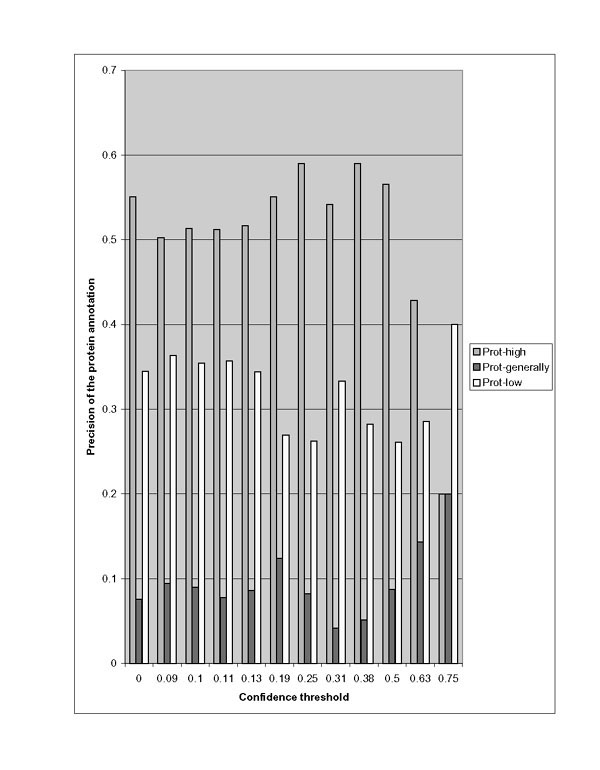
Results of the GO annotation regarding the protein (Y axis) for different levels of confidence (X axis).

**Figure 3:**
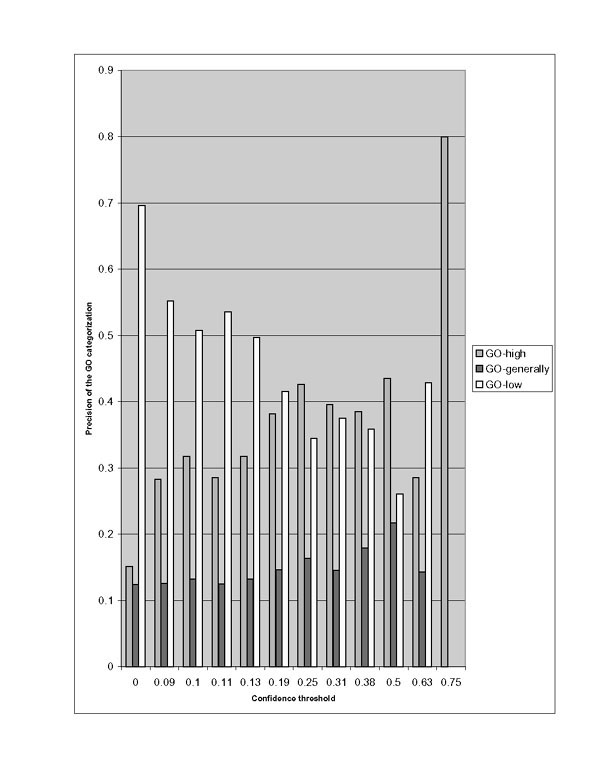
Results of the GO annotation (Y axis) for different levels of confidence (X axis).

From a more detailed perspective, results in Figures 2 and 3 gives the precision of the tool for different confidence threshold. The higher the confidence is, the less results are submitted so that increasing the value of the threshold results in decreasing the recall of the classifier. The former figure presents the quality of the *protein-passage *association, the latter presents the quality of the *GO term-passage *association. The normalized retrieval status value of each term is used to estimate the confidence of the prediction. Official results were given for all predictions, i.e. we do not set any threshold on the confidence estimator (histograms with *confidence threshold *= 0 on the X axis). When all results are examined, our system achieved the best recall-precision ratio (comparative results can be found in Couto et al.'s report [40]) but more interesting is the fact that the retrieval status value can serve opportunely as confidence estimator. Thus, the number of *high *marks directly follows the threshold and symmetrically the number of *low *marks tends to decrease. Finally, the number of *generally *tend to be equally distributed especially on Figure 3. Results of experiments generated after the competition are reported in Table 9. The official run is also located in this table for comparison. At first sight, we see that experiments carried out after the competition were able to improve the performance of the system when measured by mean average precision and mean reciprocal rank. Compared to other evaluations campaigns, the BioCreative initiative is probably the first large-scale effort to establish user-centered results and tasks based on sound utility measures. However, we regret that the BioCreative task 2 did not deliver an evaluator-independent benchmark, that could be reused for other experiments. In this aspect, the passage retrieval task of the BioCreative task 2 clearly relates to question-answering evaluations, where results rely exclusively on human evaluations made a posteriori.

For r1, we see that the optimal weighting schema for the vector space engine (i.e. *anc.atn*) is not the best schema for combination with the regular expression pattern matcher. The best combination is achieved with *ltc.lnn *(r2). As expected the impact of the pattern matcher is especially effective at high ranks (+31.3% of MRP), while the improvement of the MAP is less significant (+19.1%). In r3, we observe that the thesaurus has a positive but marginal impact, from 15.86 to 16.10 for MRP. The submitted run (r4) confirms that linguistically-motivated phrase indexing is beneficial, from 16.06 to 16.45 for MRP and from 7.16 to 7.72 for MAP. In r4, we used *ltc.lnn*, but experiments performed after the competition time show (in r5) that a better *tf.idf *combination is *anc.ltn*. For the augmented term frequency factor, noted *a*, the value of the parameters is α = β = 0.5. Finally, the use of the GO definition to expand the document/term matching features is also beneficial (from 17.04 to 17.17 for MRP and from 8.32 to 8.61 for MAP). Run r7 uses the same settings as the official run but applied to the full articles. Although using full articles rather than abstracts results in a degradation of the classification in regards to both MAP and MRP, we cannot conclude that abstracts should be preferred to full articles. Infact, we cannot expect that the best combination for processing short abstracts would remain optimal for long articles, and therefore additional experiments with different parameters are needed to study this issue.

### Related experiments

Consistent with conclusions drawn from task 1b, is the fact that data-poor retrieval string matching methods [41] are competitive with more complex data-intensive approaches [42]. The impact of features appearing in GO definitions and related resources, which were used by some of the competitors, appear to be promising extentions [32]. Such expansion strategies could improve both the categorization and the passage retrieval task, and we believe that further experiments are necessary to fully exploit these resources. Another important evaluation parameter is the size of the retrieved passage. Guidelines were not explicit and therefore some participants [39] decided to return document sections rather than sentences. The precision (*high *results) was not improved, but they retrieved a large number of results in the *generally *category. Furthermore, it is very interesting to analyse the way these other participants envisage the relationship between tasks 2.1 and 2.2. Passage retrieval (task 2.1) is seen as a feature reduction step, which is preparatory for the GO annotation task (task 2.2). Working with full text articles, other systems must first reduce the categorization space to a shorter passage, then, categorization is applied. This design is opposite to ours. Categorization is performed first, then passage retrieval is accomplished driven by the GO category. Such strictly inverted strategies suggest that a wide span of approaches can be equally effective. However, considering a related task proposed last year in the context of the TREC Genomics track (automatic extraction of GeneRIFs in LocusLink), it seems that passages longer than a sentence are generally not appropriate for protein annotation. Thus, for the TREC Genomics track [43], Ruch et al. [27] report that sentence shortening was an effective strategy to model GeneRIF extraction as performed by humans. Finally, recent advances in Text Mining applied to biomedical litterature suggest that argumentative content [44], i.e. paragraphs or sentences specific to categories such as *purpose*, *methods*, *results *and *conclusion *might be of interest for information retrieval [45] and extraction of gene and protein functions [27].

## Conclusion

We have reported on the development and evaluation of a passage retrieval tool used to support an automatic text categorization tool for protein annotation. For passage retrieval, the tool combines an exact match strategy and a string-to-string edit distance to select the best ranked sentence. For text categorization, the systems combines: 1) a pattern matcher, based on regular expressions; 2) a vector space retrieval engines that uses stems and phrases as indexing units, a traditional *tf.idf *weighting schema, and cosine as normalization factor. The use of noun phrases seems to improve the categorization's average precision by at least 3%. The combined system can be applied on any controlled vocabulary, even when manually annotated data are not available. The system achieved very competitive results in the context of the BioCreative challenge.

## Figures and Tables

**Table 1:** GO term per record in DSI.

# GO term	# Swiss-Prot record	Proportion (%)	Total (%)
2	155	25.3	25.3
3	147	24.0	49.4
1	146	23.8	73.3
4	74	12.1	85.4
5	32	5.23	90.6
6	22	3.60	94.2
7	13	2.12	96.3
8	7	1.14	97.5
9	5	0.81	98.3
12	3	0.49	98.8
10	3	0.49	99.3
33	1	0.16	99.5
11	1	0.16	99.6
14	1	0.16	99.8
15	1	0.16	100

**Table 2:** Distribution of token per terms in the Gene Ontology.

# token	# GO term	Proportion (%)	Total (%)
1	391	2.34	2.34
2	4046	24.2	26.5
3	6263	37.5	64.1
4	2723	16.3	80.4
5	1563	9.36	89.8
6	833	4.99	94.8
7	395	2.36	97.1
8	204	1.22	98.3
9	97	0.58	98.9
10	42	0.25	99.2
11	31	0.18	99.4
12	38	0.22	99.6
13	16	0.09	99.7
14	12	0.07	99.8
15	11	0.06	99.8
16	5	0.02	99.9
17	2	0.01	99.9
19	1	0.00	99.9
22	1	0.00	99.9
24	2	0.01	99.9
25	4	0.02	99.9
26	2	0.01	99.9
27	1	0.00	99.9
28	1	0.00	99.9

**Table 3:** Example of distances for task 2.1.

Sentence	Direct match	Smith-Waterman	Levenshtein	Jaccard	Jaro	FinalScore
S1	1	19	-45	0.062	0.62	29
S2	2	51	-18	0.12	0.58	71
S3	2	18	-12	0.083	0.58	38

**Table 4:** Term Weights in the SMART System.

Term Frequency	
First Letter	*f*(*tf*)

n (natural)	*tf*
l (logarithmic)	1 + *log*(*tf*)
a (augmented)	α + β × (*tf/max*(*tf*))

Inverse Document Frequency	

Second Letter	*f*(1/*df*)

n(no)	1
t(full)	*log*(*N */*df*)

Normalization	

Third Letter	*f*(*length*)

n(no)	1
c(cosine)	

**Table 5:** Sample of GO synonyms for each axis.

function: cholesterol O-acyltransferase – sterol O-acyltransferase activity
component: protoplasm – intracellular
process: cell division – cytokinesis

**Table 6:** Sample of GO definitions.

term: TRAIL receptor 2 biosynthesis
goid: GO:0045559
definition: The formation from simpler components of TRAIL-R2 (TNF-related apoptosis inducing ligand receptor 2), which engages a caspase-dependent apoptotic pathway and mediates apoptosis via the intracellular adaptor molecule FADD/MORT1.
term: trans-2-enoyl-CoA reductase (NADPH) activity
goid: GO:0019166
definition: Catalysis of the reaction: acyl-CoA + NADP+ = trans-2,3-dehydroacyl-CoA + NADPH + H+.

**Table 7:** Distribution of the most frequent GO terms in the 640 items Swiss-Prot data set (DSI): cut-off at 14 occurrences.

GO ID	# Occurence	Proportion (%)	Total (%)	Term
GO:0005634	62	3.41	3.41	nucleus
GO:0007165	58	3.19	6.60	signal transduction
GO:0005737	50	2.75	9.36	cytoplasm
GO:0005887	47	2.58	11.9	integral to plasma membrane
GO:0005886	30	1.65	13.6	plasma membrane
GO:0003700	27	1.48	15.0	transcription factor activity
GO:0016021	27	1.48	16.5	integral to membrane
GO:0005515	19	1.04	17.6	protein binding
GO:0006412	16	0.88	18.5	protein biosynthesis
GO:0006810	15	0.82	19.3	transport
GO:0006468	14	0.77	20.0	protein amino acid phosphorylation

**Table 8:** Passage retrieval: results for each GO axis.

	biological process	cellular component	molecular function	Total
# submitted passage	710	185	361	1256

# evaluated passage	330	126	205	661

GO-high	12 %	20 %	18 %	15 %
GO-generally	11 %	09 %	16 %	12 %
GO-low	74 %	67 %	64 %	70 %

Prot-high	59 %	55 %	49 %	55 %
Prot-generally	05 %	05 %	13 %	08 %
Prot-low	33 %	36 %	36 %	35 %

**Table 9:** Results for different system settings.

Run	VS	RegEx	Thesaurus	NP	GO Definition + Prior	Full Article	MRP (*δ *%)	MAP (*δ *%)
r1 (baseline)	anc.atn	-	-	-	-	-	12.08	5.97
r2	ltc.lnn	x	-	-	-	-	15.86 (+31.3)	7.11 (+19.1)
r3	ltc.lnn	x	x	-	-	-	16.10 (+33.2)	7.34 (+22.5)
r4 (official)	ltc.lnn	x	x	x	-	-	*16.51 (+36.7)*	*7.85 (+31.4)*
r5	anc.ltn	x	x	x	-	-	16.89 (+39.8)	8.02 (+34.3)
r6	anc.ltn	x	x	x	x	-	*17.10 (+41.5)*	*8.11 (+35.8)*
r7	ltc.lnn	x	x	x	-	x	16.02 (+32.6)	7.34 (+22.9)
